# Radiological identification and analysis of soft tissue musculoskeletal calcifications

**DOI:** 10.1007/s13244-018-0619-0

**Published:** 2018-06-07

**Authors:** Véronique Freire, Thomas P. Moser, Marianne Lepage-Saucier

**Affiliations:** Department of Radiology, Radio-Oncology and Nuclear Medicine, Centre hospitalier de l’Université de Montréal, 1000 rue Saint-Denis, Montréal, QC H2X 0C1 Canada

**Keywords:** Calcium, Tendinopathy, Arthritis, Radiography, Imaging

## Abstract

**Abstract:**

Musculoskeletal calcifications are frequent on radiographs and sometimes problematic. The goal of this article is to help radiologists to make the correct diagnosis when faced with an extraosseous musculoskeletal calcification. One should first differentiate a calcification from an ossification or a foreign body and then locate the calcification correctly. Each location has a specific short differential diagnosis, with minimal further investigation necessary. Intra-tendon calcifications are most frequently associated with hydroxyapatite deposition disease (HADD). In most cases, intra-articular calcifications are caused by calcium pyrophosphate dihydrate (CPPD) crystal deposition disease. Soft tissue calcification can be caused by secondary tumoural calcinosis from renal insufficiency, or collagen vascular diseases and by vascular calcifications, either arterial or venous (phlebolith).

**Teaching Points:**

•* Calcifications have to be differentiated form ossification and foreign body.*

•* A musculoskeletal MRI study must always be correlated with a radiograph.*

•* The clinical manifestations of calcifications may sometimes mimic septic arthritis or sarcoma.*

•* HADD and CPPD crystal deposition have a distinct appearance on radiograph.*

•* Calcinosis is more frequently caused by chronic renal failure and scleroderma.*

## Introduction

Soft tissue musculoskeletal calcifications are seen on radiographs on a daily basis. Oftentimes, the radiologist is uncomfortable regarding how to report them or if further investigations is necessary. While seemingly trivial, calcifications can be an early indication of an unsuspected pathology.

Two categories of calcifications are recognised: dystrophic and metabolic (also termed metastatic) [1]. Dystrophic calcifications occur in necrotic or damaged tissue with normal serum levels of calcium and may progress to ossification. They represent more than 95% of calcifications observed in radiology. Metastatic calcifications are generally diffuse, occur in otherwise normal tissue, and are associated with abnormal serum levels of calcium, phosphate (increased calcium-phosphate product) and other ions [2].

**Fig. 1:**
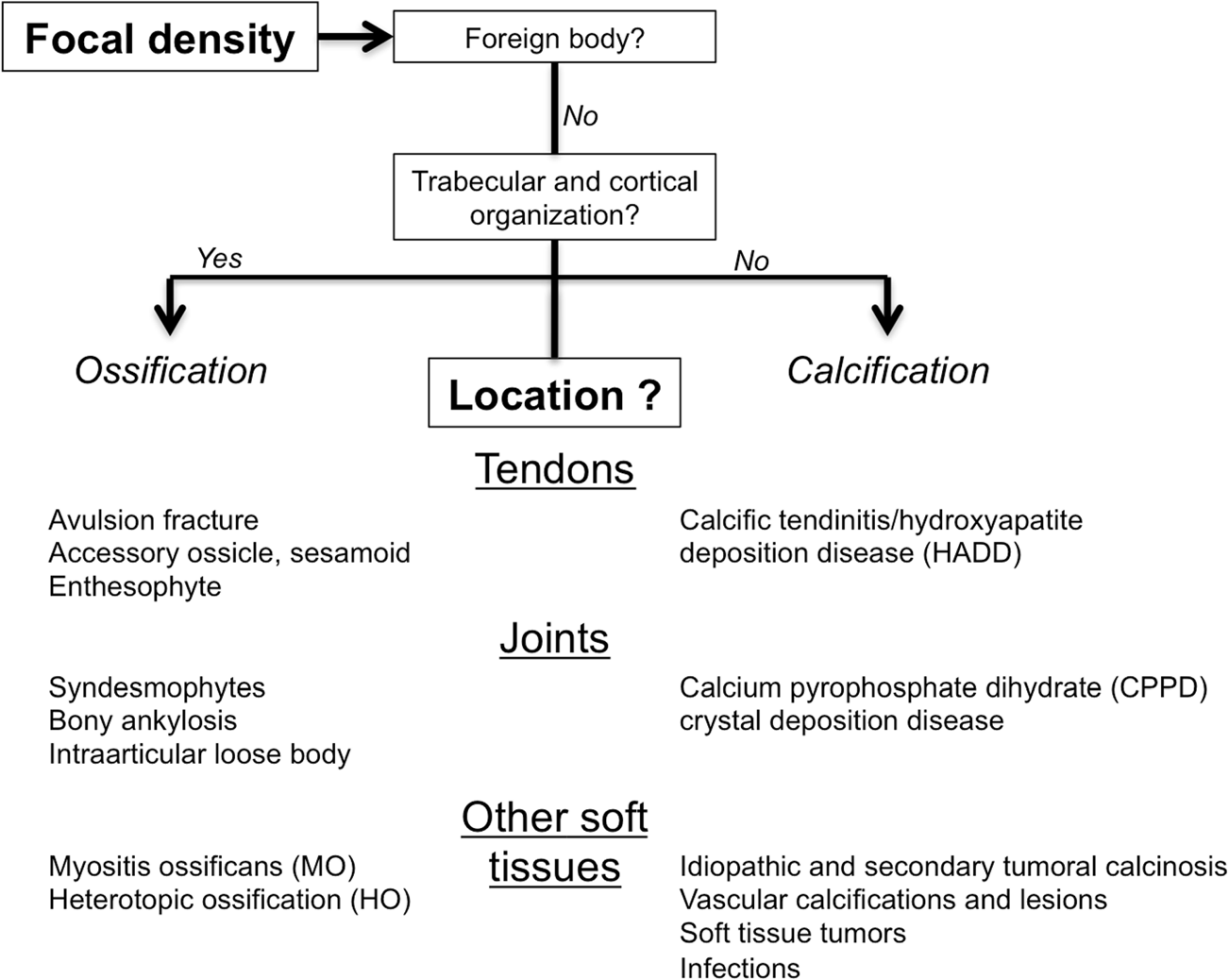
Flow chart shows approach to radiographic evaluation of focal density

Calcifications have multiple appearances, locations and causes. With a careful and systematic analysis of their characteristics, it is often possible to narrow the differential diagnosis, sometimes with minimal further investigations. Therefore, in this article, instead of classifying calcifications by their aetiology, we propose a more practical approach based on location, information available to the radiologist. We will describe our two-step method regarding musculoskeletal densities on radiographs: firstly, the correct identification of a calcification, and secondly, its location (Fig. 1). Tips to differentiate one diagnosis from another will be discussed.

## First step: Is it really a calcification?

The first step is to differentiate calcifications from ossifications or foreign bodies. One can achieve this goal by analysing its density, shape and pattern.

### Density

**Fig. 2:**
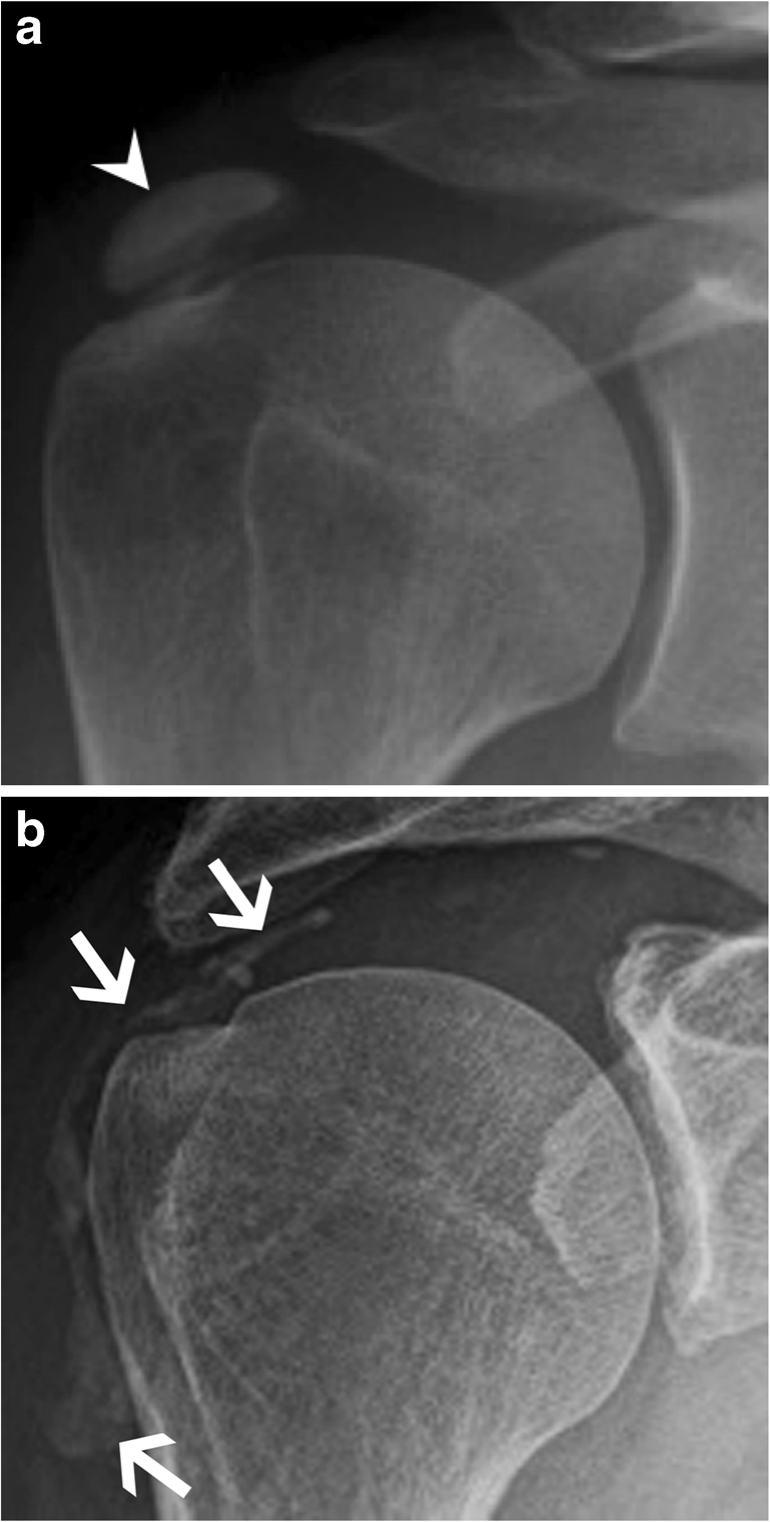
HADD. (**a**) Anteroposterior radiograph of the right shoulder shows a calcific tendinopathy of the supraspinatus tendon (arrowhead) during formative or resting phase. (**b**) Anteroposterior radiograph of the right shoulder in a different patient shows migration of the calcifications to the subdeltoid-subacromial bursa (arrows) during the resorptive phase

**Fig. 3:**
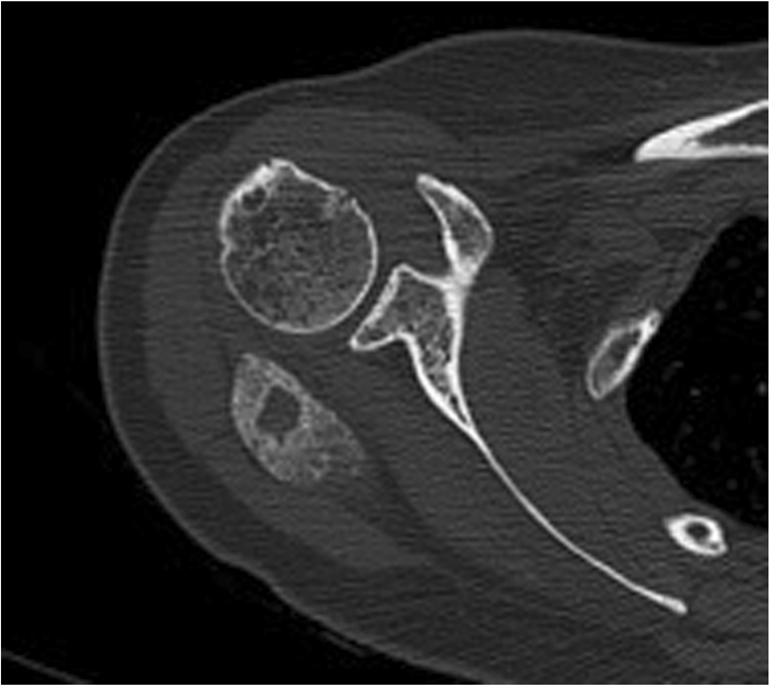
Ossification. Axial CT image of myositis ossificans in the infraspinatus muscle shows a cortical and trabecular mineralisation pattern with a zonal distribution (absence of mineralisation centrally due to fatty bone marrow)

**Fig. 4:**
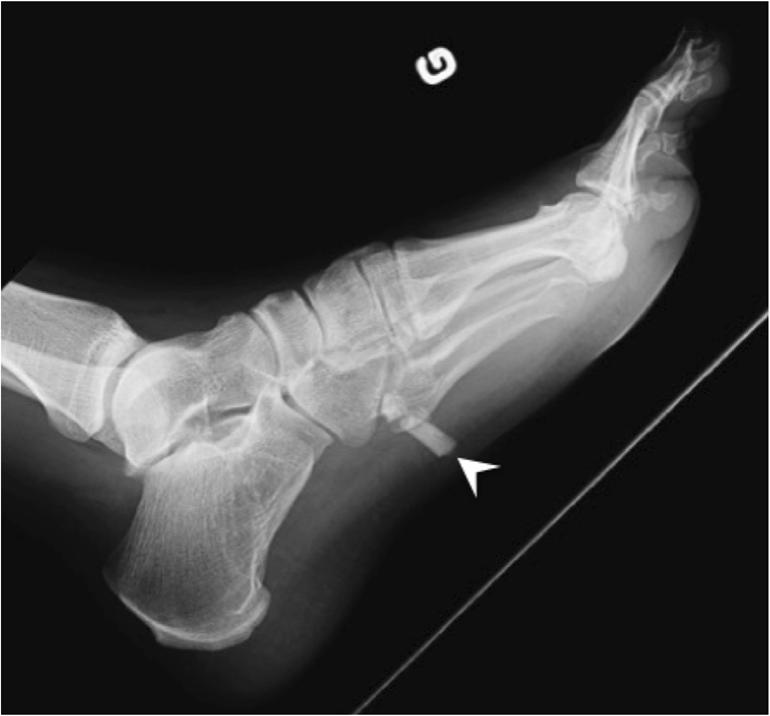
Foreign bodies. Lateral radiograph of the foot shows glass fragments (arrowhead) in the plantar soft tissues, with sharp edges and a rectangular shape

Calcifications normally appear as mineralised densities with an attenuation coefficient higher than soft tissue but lower than bone (Fig. 2). With computed tomography (CT), there is a wide range of Hounsfield unit (HU) values for calcifications, but it is mostly between 100 and 400 HU, whereas bone reaches higher values (700 HU for trabecular bone and over 1500 HU for cortical bone; Fig. 3). Foreign body density varies depending on the nature of the material, but glass and silicone, among others, may have similar HU values to calcifications (Fig. 4) [3].

CT is more sensitive than radiography for the detection and analysis of calcifications and provides additional information regarding adjacent tissues such as neurovascular bundles, muscles, tendons and inflammatory reactions [4]. Magnetic resonance imaging (MRI) shows various signal intensities on conventional spin echo T1- or T2-weighted images and can frequently overlook calcifications, hence the importance to correlate MRI with conventional radiographs on every occasion. Misleading appearances can occur such as fluid signal (mimicking tear) or intense inflammatory reaction surrounding a calcification. Gradient echo sequences and, more recently, a three-dimensional fast low-angle gradient-recalled echo (GRE) sequence (susceptibility-weighted imaging [SWI]) can depict calcifications more accurately. Based on phase images, the latter allows a better distinction between calcifications and other causes of susceptibility variations [5]. Ultrasound is known to be more sensitive than radiography, however, when acoustic shadowing is present, it will prevent one from doing a complete assessment and being able to differentiate between ossification and calcification [6].

### Shape and patterns

**Fig. 5:**
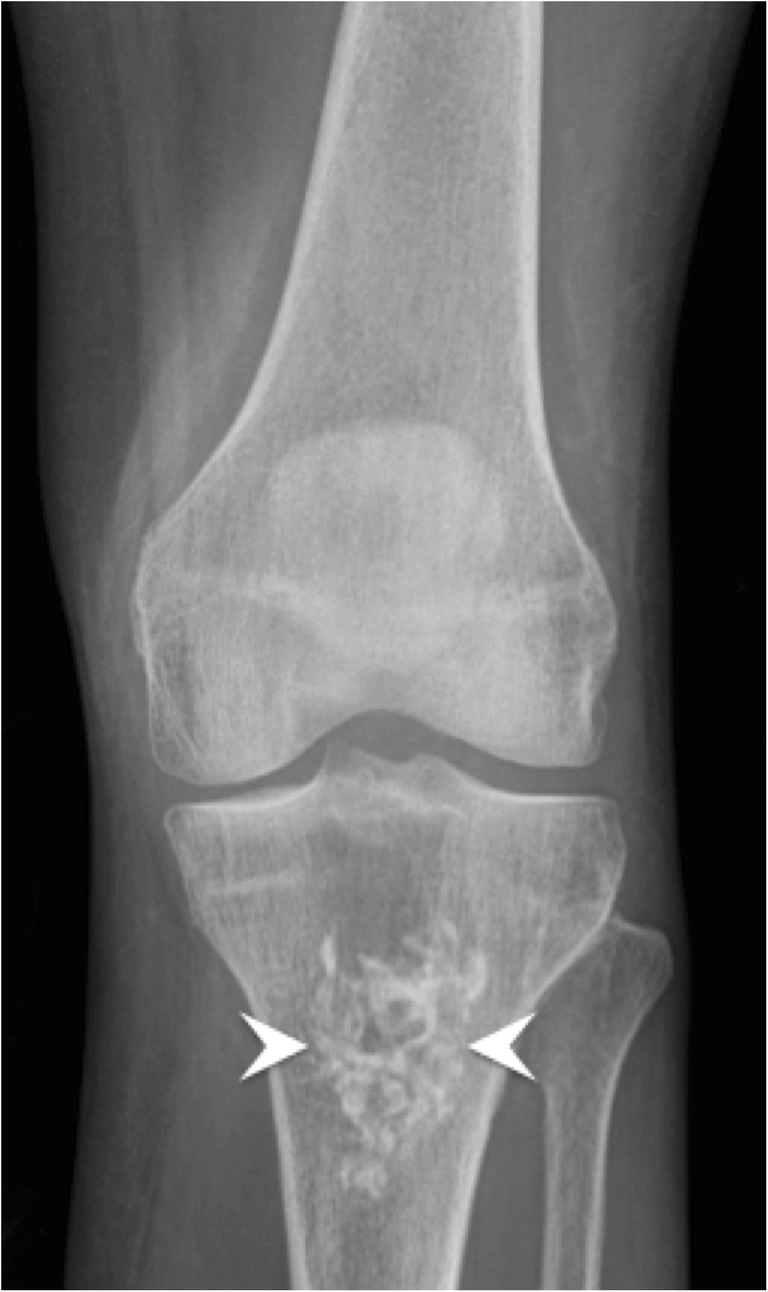
Enchondroma. Anteroposterior radiograph of the left knee shows a typical chondroid matrix mineralisation with arcs and rings in the tibia (arrowheads)

The classic hydroxyapatite calcification has an amorphous and cloudlike appearance with a well-defined oval contour (Fig. 2) [7]. It can appear multiloculated and even show fluid-fluid levels. Linear calcifications can be related to calcium pyrophosphate dihydrate (CPPD) crystal deposition. Punctate calcifications with arcs and rings will be seen in cartilage-forming tumours and are referred to as cartilaginous matrix mineralisation (Fig. 5) [8].

On the other hand, radiographs of ossifications will show a bone organisation with a distinct cortical and/or trabecular bone pattern (Fig. 3). Fatty bone marrow within trabecular spaces can be identified on CT and MRI (Fig. 3), narrowing greatly the differential diagnosis (Table 1). However, immature ossifications are not so well organised and may be more difficult to diagnose [2].

**Table 1:** Differential diagnosis of calcifications and ossifications according to their location

Location	Calcification	Ossification
Tendon	**HADD calcific tendinitis** CPPD deposition disease	**Enthesophyte** Bone avulsion fracture **Accessory bone/Sesamoid** **Tendon rupture sequela** Seronegative arthropathy (proliferative enthesitis)
Articular	**CPPD deposition disease** Debris from destructive arthropathy (Charcot joint, RDO) HADD (ex: facet joint, crowned dens syndrome) Synovial chondromatosis Corticoid injection Gout with tophus mineralisation	Ankylosis Syndesmophytes **Heterotopic ossification** (underlying DISH? Surgery? Neuropathy?)
Other soft tissues	Idiopathic tumoural calcinosis **Secondary calcinosis** (**CRF**, SSc) Soft tissue tumours (lipoma, chondroma, nerve sheath tumours, synovial sarcoma, other extraosseous sarcomas) **Vascular calcifications**: atherosclerosis, phlebolith Infections, lymph nodes	MOC **Heterotopic ossification** (burns?)

The most frequent aetiologies are in bold characters. *CPPD*, calcium pyrophosphate dihydrate; *CRF*, chronic renal failure; *DISH*, diffuse idiopathic skeletal hyperostosis; *HADD*, Hydroxyapatite deposition disease; *MOC*, myositis ossificans circumscripta; *RDO*, rapidly destructive osteoarthritis; *SSc*, progressive systemic sclerosis

Foreign bodies will often show a distinctive shape such as sharp and/or geometric borders helping identifying their nature (Fig. 4). Other patterns can be recognised—such as round calcifications with radiolucent centre in venous phleboliths; parallel lines in calcifications of arterial origin; or “rice-grain” calcification in parasitic infection—and will be discussed later.

## Second step: What is the exact location?

Once a calcification is recognised on a radiograph, its location can be determined when its shape follows that of the anatomical structure involved, or more routinely using multiple oblique views around joints. CT can be helpful but is most often not necessary. Table 1 summarises the differential diagnosis of calcifications and ossifications based on their location.

### Tendon calcifications

#### Calcific tendinopathy (hydroxyapatite deposition disease, HADD)

Calcific tendinopathy is very frequent and seen in 3–15% of the general population [9–11]. It peaks in the fifth decade, can be bilateral in up to 50% of patients and symptomatic in 10–50% of cases [12]. It is thought to be the result of hydroxyapatite deposition disease (HADD) that accumulates in degenerated or traumatised tendons through a process of fibrocartilaginous metaplasia [13, 14].

Successive stages have been described in the literature and may help in understanding the various imaging appearances of calcific tendinopathy. The most practical classification, by Uhthoff, separates pre-calcific, calcific and post-calcific stages [14]. The calcific stage is further divided into formative, resting and resorptive phases.

**Fig. 6:**
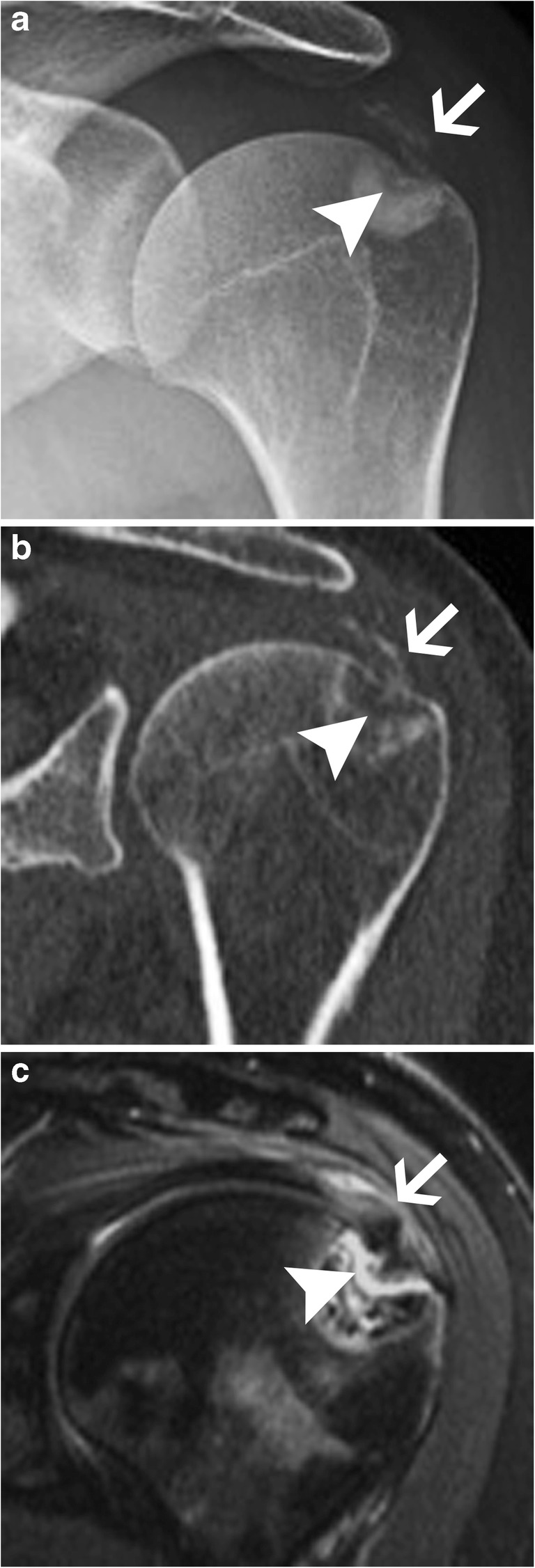
Supraspinatus calcifying tendinopathy with intraosseous extension. (**a**) Frontal radiograph, (**b**) coronal reformat CT image and (**c**) coronal fat-saturated T2-weighted image show supraspinatus amorphous calcifications (arrows) with intraosseous extension causing erosions (arrowheads). Note the sclerosis around the erosion, hyperdense in (**a**) and (**b**) and hypointense in (**c**)

The formative and resting phases are associated with a dense, homogeneous and well-defined calcium deposit (Fig. 2a). They can be asymptomatic or present with a mild to moderate degree of discomfort caused by impingement of a bulky calcification. The resorptive phase is characterised clinically by acute, sometimes excruciating, pain with the release and migration of calcium in surrounding tissues, bursae, joints or even bones. On radiographs, the calcification becomes fluffy, ill-defined (including a comet tail appearance) and less dense or even unapparent. The intra-bursal migration of calcifications can be seen as a dense crescent streak overlying the main calcification (Fig. 2b) [13, 15]. This resorptive phase can have misleading appearances on imaging, including bone erosions on radiographs, bone marrow oedema on MRI or bone uptake on nuclear medicine studies. Intraosseous resorption can classically be mistaken for infection or tumour, hence the importance of identifying the continuity between the erosion and the calcific tendinitis (Fig. 6) [16]. Clinically, the resorptive phase can mimic pseudoparalysis, a septic joint or a fracture, hence the importance of acquiring a radiograph and avoiding unnecessary joint aspiration or even arthrotomy. Despite the spontaneously favourable outcome, image-guided treatment can be considered in cases of refractory pain after conservative treatment (analgesics, NSAIDs, rest and physiotherapy), including ultrasound (US)-guided aspiration and/or injection of anaesthetic with a corticoid into the surrounding tissue, more frequently in the subacromial subdeltoid bursa, avoiding intra-tendinous injection [17]. The post-calcific stage will show either a residual shell, linear calcification remnant, or a complete resolution.

**Fig. 7:**
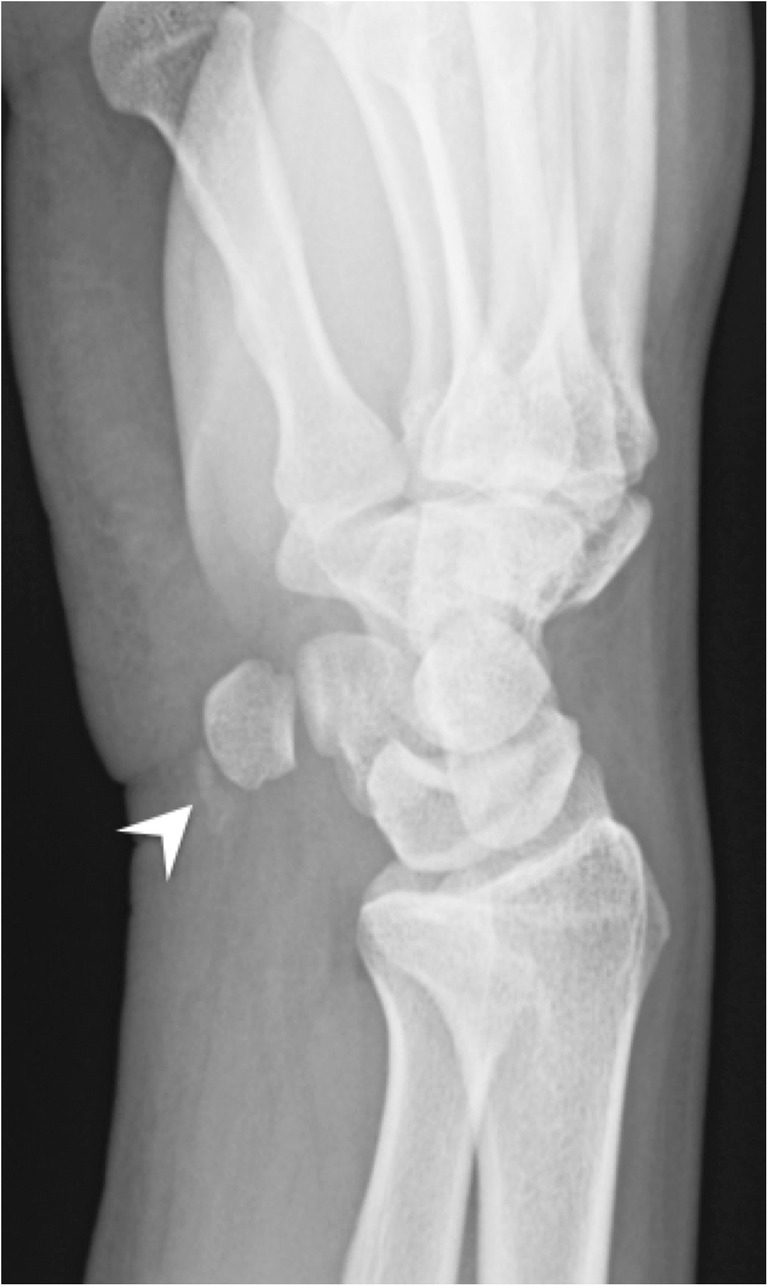
Calcifying tendinopathy of flexor carpi ulnaris. Oblique radiograph of the wrist shows HADD in the flexor carpi ulnaris with amorphous cloudlike calcifications (arrowhead)

Calcific tendinopathy is seen most frequently at the shoulder (Fig. 2), especially in the supraspinatus tendon, followed by the wrist, the hip and the elbow, but virtually every tendon can be involved (Fig. 7) [13, 15]. The radiologist should always describe calcifications of HADD, giving the location (tendon involved), the size and the appearance (well-delineated or ill-defined, the latter being more often symptomatic) for comparison with follow-up studies.

#### Non-HADD tendon mineralisation

**Fig. 8:**
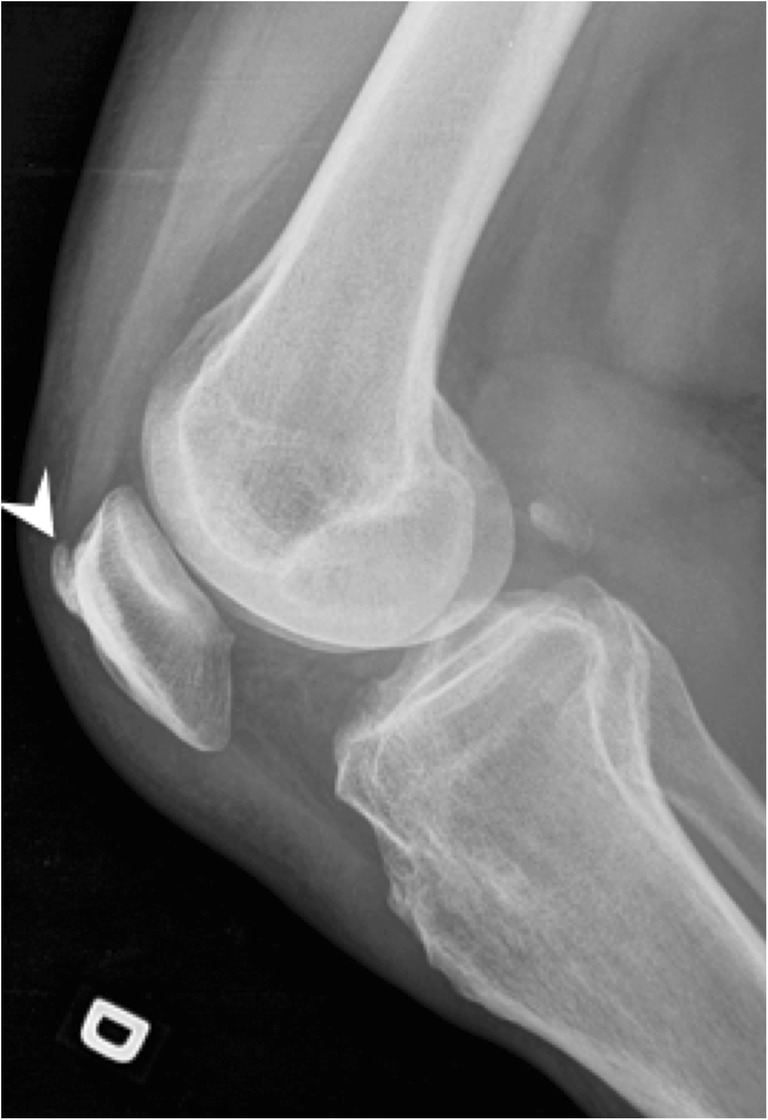
Enthesophyte. Lateral radiograph of the right knee shows an ossified enthesophyte at the insertion of the quadriceps tendon on the patella (arrowhead)

One must differentiate calcific tendinopathy from degenerative enthesopathy seen at the insertion of the tendon to the bone (enthesis). Those calcifications appear more often with aging and do not show resolution, as opposed to the calcific tendinopathy. The enthesopathy can progress to coarser ossification (Fig. 8). Ossification of entheses is also a feature of seronegative arthritides such as psoriasis, ankylosing spondylitis or reactive arthritis, as well as diffuse idiopathic skeletal hyperostosis (DISH). Finally, intratendinous ossifications can occur following injury or surgery, for example in the Achilles tendon and will show a typical bone pattern organisation.

**Fig. 9:**
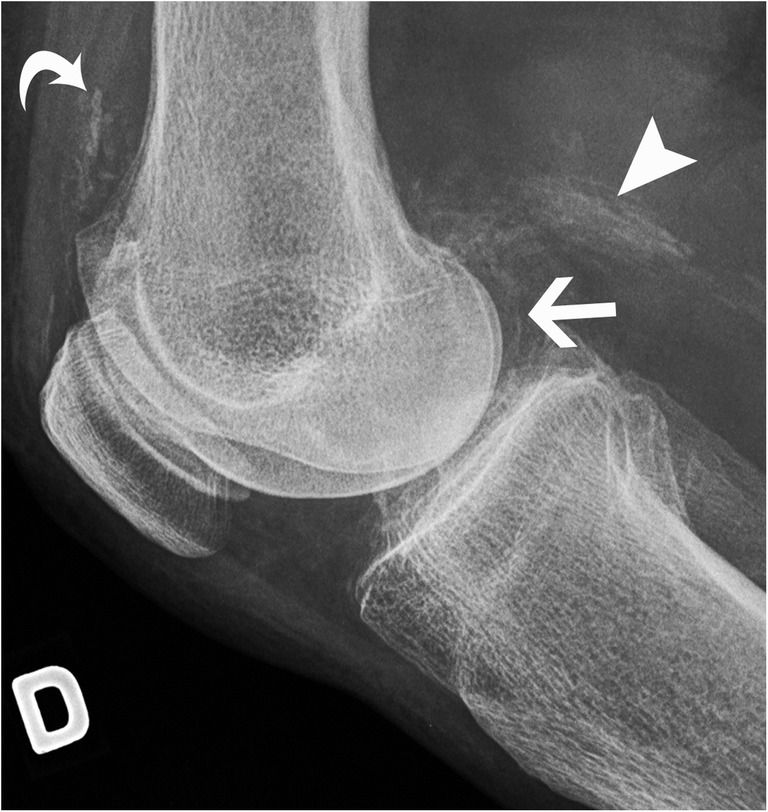
CPPD crystal deposition disease in the knee. Lateral radiograph of the right knee shows chondrocalcinosis in hyaline cartilage at the posterior femoral condyle (arrow), in the gastrocnemius proximal tendon (arrowhead) and in the synovial lining at the suprapatellar recess (curved arrow)

Calcium pyrophosphate dihydrate (CPPD) crystals deposition can occur in tendons and appear linear and delicate, sometimes stratified [18]. Identifying chondrocalcinosis in a nearby joint will help the radiologist to reach the correct diagnosis (Fig. 9) [19, 20]. Table 1 highlights the differential diagnosis of tendon mineralisation by dividing the pathologies with calcifications from the ones with ossifications.

### Articular calcifications

#### Calcium pyrophosphate dihydrate (CPPD) crystal deposition disease

It is the archetype of articular calcifications and most frequent cause of crystal-induced arthropathies [21]. There is confusion around terms like chondrocalcinosis and pseudogout, which needs clarification. Chondrocalcinosis is a descriptive term referring to the presence of visible calcifications in cartilage tissue (at imaging or on microscopy) and does not refer to any clinical syndrome per se [18]. On the other hand, “pseudogout” is a term referring to a clinical scenario of acute arthritis mimicking a gout attack, hence the name pseudogout. CPPD crystal deposition disease is the accepted term and refers to both chondrocalcinosis and CPPD arthropathy. An increase of the intra-articular concentration of extracellular inorganic pyrophosphates is believed to cause CPPD arthropathy and this is the result of an abnormality of the local metabolism of the synovial fluid and probably also of the articular cartilage. The crystal deposition in the joint leads to calcification accumulation in the articular cartilage, which can be seen at imaging [22]. The sporadic form is the most frequent, but one must keep in mind rare hereditary cases or secondary causes such as haemochromatosis, hyperparathyroidism and other entities, which are beyond the scope of this article. These secondary conditions all show an increase of the intra-cellular CPP crystal deposition by various mechanisms.

On imaging, the calcification is seen in hyaline cartilages and fibrocartilages (menisci, acetabular labrum, pubic symphysis, intervertebral discs), but also in ligaments, capsules and tendons. In hyaline cartilage, it parallels the subchondral bone (Fig. 9). In soft tissues, it has a delicate linear and/or stratified appearance—as opposed to the usual nodular and discrete appearance of HADD—and occurs in an older population. Chondrocalcinosis is very frequent in the elderly asymptomatic population, reaching possibly 45% of the population aged 85 years old or above [21, 23]. It is seen most frequently in the knee, followed by the wrist, pubic symphysis and the hip [21]. In fact, when chondrocalcinosis is identified without any symptoms or signs of arthropathy in an elderly patient, it is usually considered an irrelevant finding with no clinical significance.

**Fig. 10:**
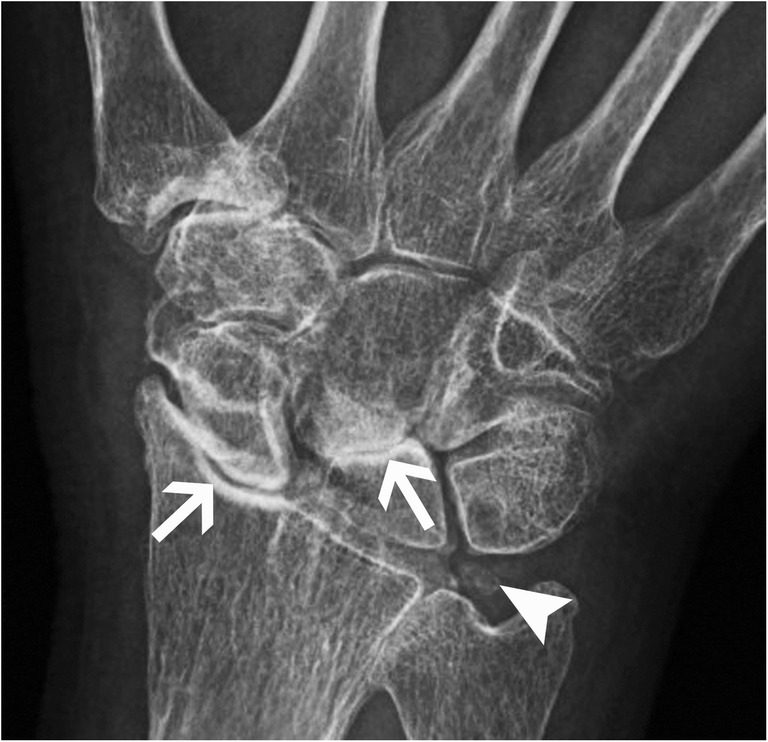
CPPD arthropathy of the wrist. Posteroanterior radiograph of the wrist shows joint space narrowing and dense subchondral sclerosis at the radioscaphoid and lunocapitate joints (arrows) associated with widening of the scapholunate space, consistent with a SLAC wrist. Note chondrocalcinosis at the ulnocarpal joint space within the triangular fibrocartilage (arrowhead)

However, when there is chronic CPPD arthropathy, it resembles osteoarthritis but harbours distinctive features, such as small or absent osteophytes, well-defined subchondral sclerosis and large subchondral cysts. Its distribution is also different from osteoarthritis, with more frequent involvement of the patellofemoral joint; the radiocarpal joint associated with a scapholunate advance collapse (SLAC) appearance [24, 25]; the second and third metacarpophalangeal joints; and the glenohumeral joint (Fig. 10).

**Fig. 11:**
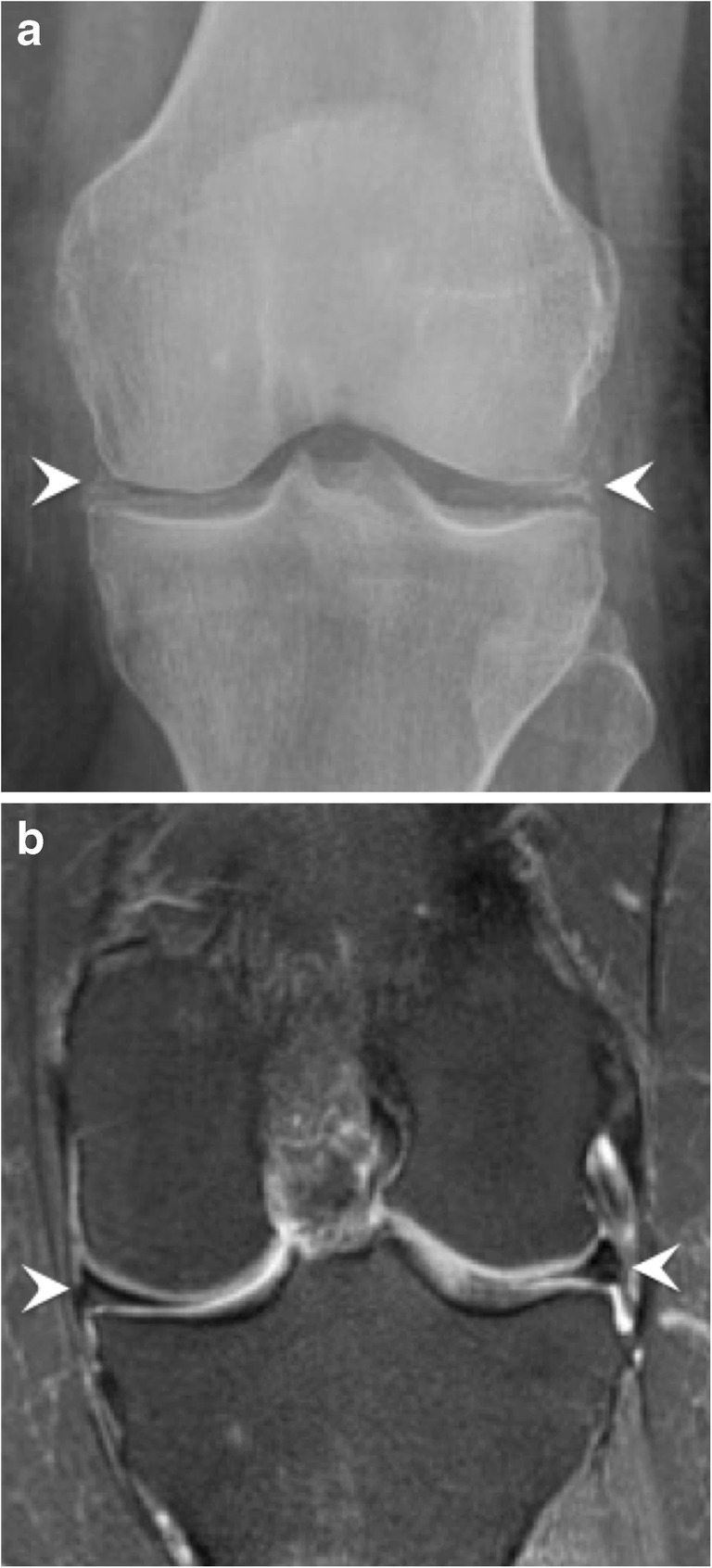
Chondrocalcinosis in the knee. (**a**) Anteroposterior radiograph of the left knee and (**b**) coronal fat-saturated proton density (PD)-weighted MRI image show chondrocalcinosis in the menisci (arrowheads)

The recommended radiographic workup for suspected CPPD arthropathy is: anteroposterior (AP) view of the knees; posteroanterior view of the wrists; and AP view of the pelvis, in order to look for chondrocalcinosis and a distinctive degenerative joint disease pattern [21]. On MRI, chondrocalcinosis will show low signal intensities in the hyaline cartilage, better seen with gradient-echo sequences [18]. The differential diagnosis for this pattern is hemosiderin deposits from trauma or haemophilia; gas related to vacuum effect; and susceptibility artefacts due to post-surgical metallic debris. Meniscal chondrocalcinosis can mimic a meniscal tear, once again showing the importance of performing radiographs with all MRI studies (Fig. 11) [26].

#### Other articular or peri-articular calcifications

**Fig. 12:**
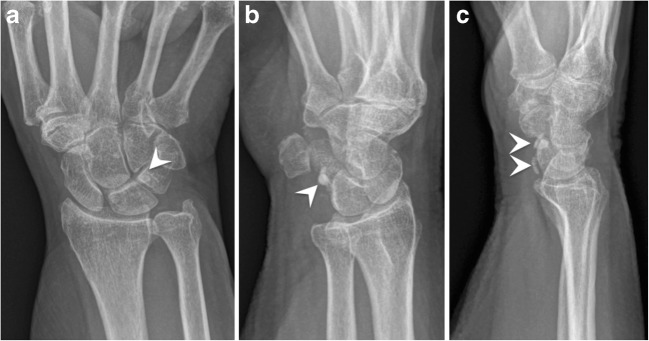
Periarticular calcifications following corticoid injection. (**a**) Frontal, (**b**) oblique and (**c**) lateral radiographs of the wrist show palmar soft tissue calcifications (arrowhead) following carpal tunnel injection of corticoid

HADD can also be found in periarticular tissues such as capsule and ligaments, presenting the same characteristics of tendinous calcifications: a well-delineated oval-shaped amorphous density in the resting phase with modifications during the resorptive phase. The differential diagnosis for intra-articular and peri-articular calcification includes recent corticoid injection (Fig. 12) [27].

##### Specific case of articular calcifications combined with joint destruction

**Fig. 13:**
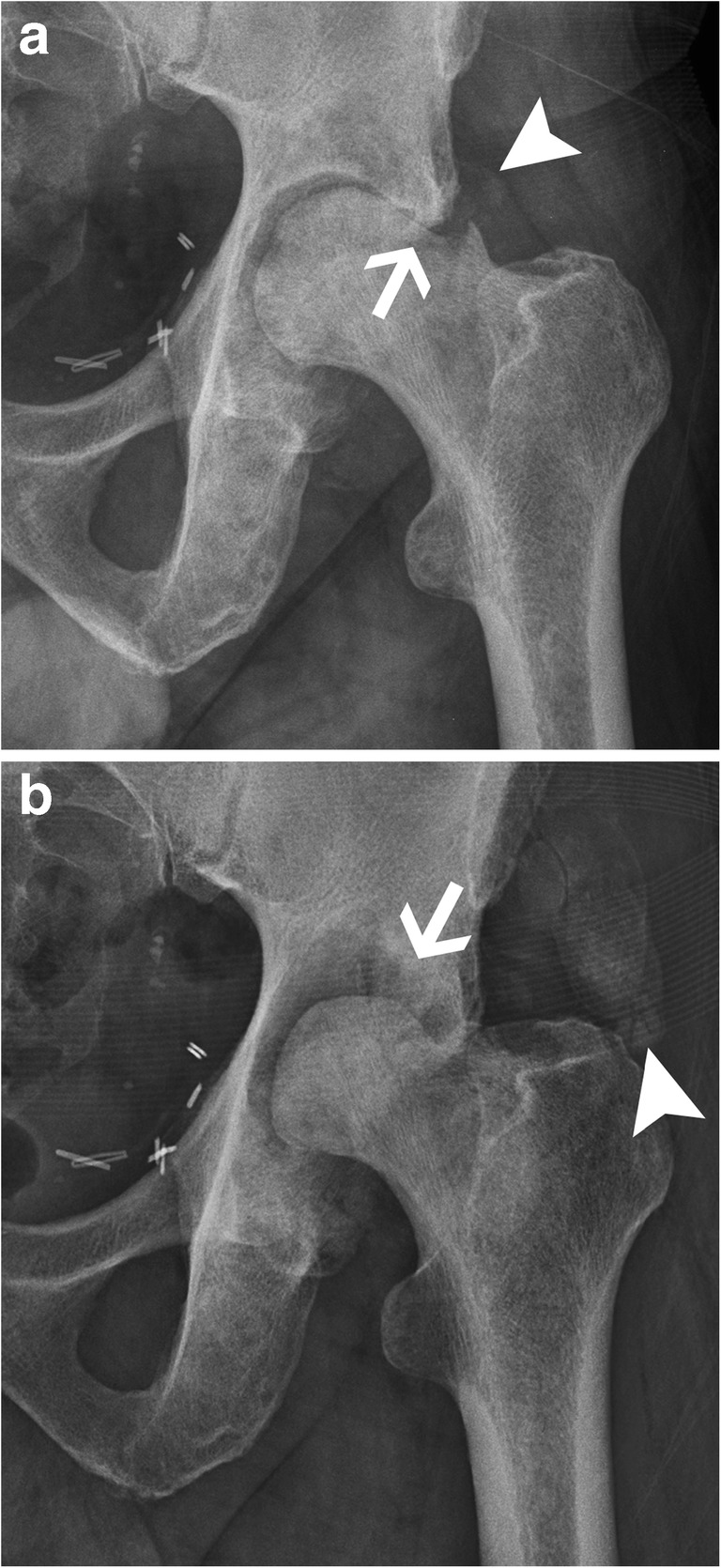
Rapid degenerative arthropathy. (**a**) Anteroposterior radiographs initially and (**b**) 3 months later show progressive bone lysis (arrows) and periarticular calcifications (arrowheads)

Multiple names have been given to this entity: Milwaukee shoulder, Postel’s arthropathy, rapidly degenerative osteoarthritis, etc. The exact mechanism has not yet clearly been established, but crystals seem to be involved, either as a causative factor inducing a severe inflammatory response or as the results of bone destruction and subsequent release of bone crystals [28]. The common clinical feature is rapid progression of joint destruction with marked resorption of bone and small or absent osteophytes (Fig. 13) [29]. When the joint is severely destroyed, it can be useful to search for signs of CPPD deposition in other joints.

Differential diagnosis includes septic arthritis, neuropathic arthropathy and avascular necrosis with joint collapse. Absence of fever with normal blood cell count and inflammatory markers will help to exclude septic arthritis, but a Gram stain and culture of joint fluid should be undertaken since crystal synovitis can coexist with sepsis [18]. On imaging, lack of osteopenia and of focal erosion are usually found. A neuropathic joint usually occurs in a patient with known underlying neurologic disorder. These two entities must be excluded since they represent contraindication to perform joint replacement, which is the treatment of choice for rapidly degenerative osteoarthritis.

##### Specific case of crystal deposition disease in the spine

Calcifications involving the transverse ligament around the odontoid process can be seen in crowned dens syndrome, typically associated with CPPD crystal deposition [22]. Oftentimes an incidental finding in elderly patients, it can occasionally be associated with fever, neck pain and stiffness thus mimicking meningitis. Calcific tendinitis of the longus colli falls within the spectrum of HADD, clinically mimicking retropharyngeal abscess and spondylodiscitis. On imaging it is usually best seen on CT, but MRI can show a mass effect with peripheral enhancement that can be confusing if the radiologist is not aware of this entity [30].

**Fig. 14:**
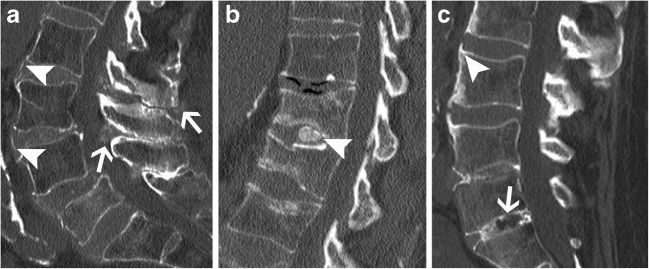
Disc calcifications. Sagittal CT images of the lumbar spine shows (**a**) calcium pyrophosphate dehydrate (CPPD) crystal deposition (arrowheads in a), (**b**) hydroxyapatite crystal deposition (arrowhead in b) and (**c**) syndesmophytes (arrowhead, in c) in three different patients. Note calcifications in ligamentum flavum and interspinous ligament associated with CPPD deposition disease (arrows in a) and ossification of disc space associated with ankylosing spondylitis (arrow in c). Note linear calcifications paralleling the vertebral endplates in b, associated with height loss, corresponding to subacute fractures in a patient with osteoporosis

Intradiscal CPPD deposition mimics syndesmophytes with thin, vertical annular calcifications [21], whereas HADD shows round and central calcifications in the nucleus pulposus (Fig. 14) [22]. The main differential is simple degenerative spondylosis, but discal calcifications and ossifications are also seen in ankylosing spondylitis or following surgery, trauma and/or infection of a disc. A diffuse pattern of disc calcification must raise suspicion for a systemic disorder causing metabolic calcifications (calcinosis) or very rarely ochronosis. A destructive spondyloarthropathy caused by crystals will typically lack osteopenia and rather show dense sclerosis and eburnation with disc space narrowing [31]. It can be associated with malalignment, subchondral fracture and “bone sand” as described by Charran et al. [31]. It can mimic infectious discitis, neuroarthropathic change, gout-related and haemodialysis-related spondyloarthropathy. Again, looking at other joints for chondrocalcinosis or typical CPPD calcification in tendons, ligaments or capsule will help in suggesting the correct diagnosis.

**Fig. 15:**
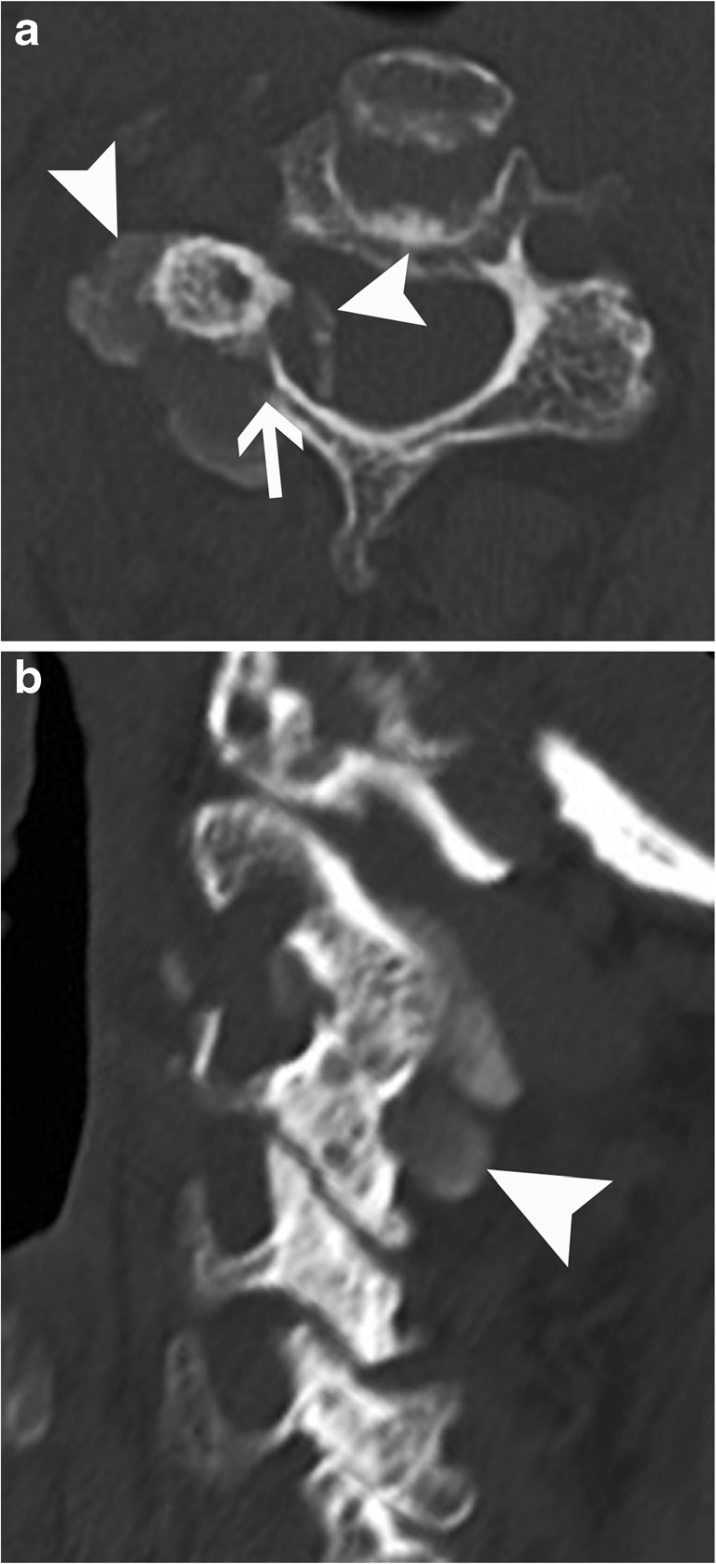
Cervical facet joint calcifications. (**a**) Axial and (**b**) sagittal CT images of the cervical spine show dense material (arrowheads) in the right C2–C3 facet joint. Note the associated bone erosion of the right lamina (arrow)

Facet joints can also be involved with HADD, presenting as an acute arthritis mimicking a septic joint. A CT investigation will more easily show the hyperdense cloudlike and amorphous intra-articular calcium deposit, than a plain radiograph or MRI (Fig. 15). MRI will show intense inflammatory reaction surrounding the facet joint in the acute symptomatic phase, sometimes with hypointense material. CT-guided aspiration might be necessary to rule out septic arthritis. As already mentioned, the differential diagnosis would be recent intra-articular steroid injection.

### Differential diagnosis of articular calcifications

#### Gout

**Fig. 16:**
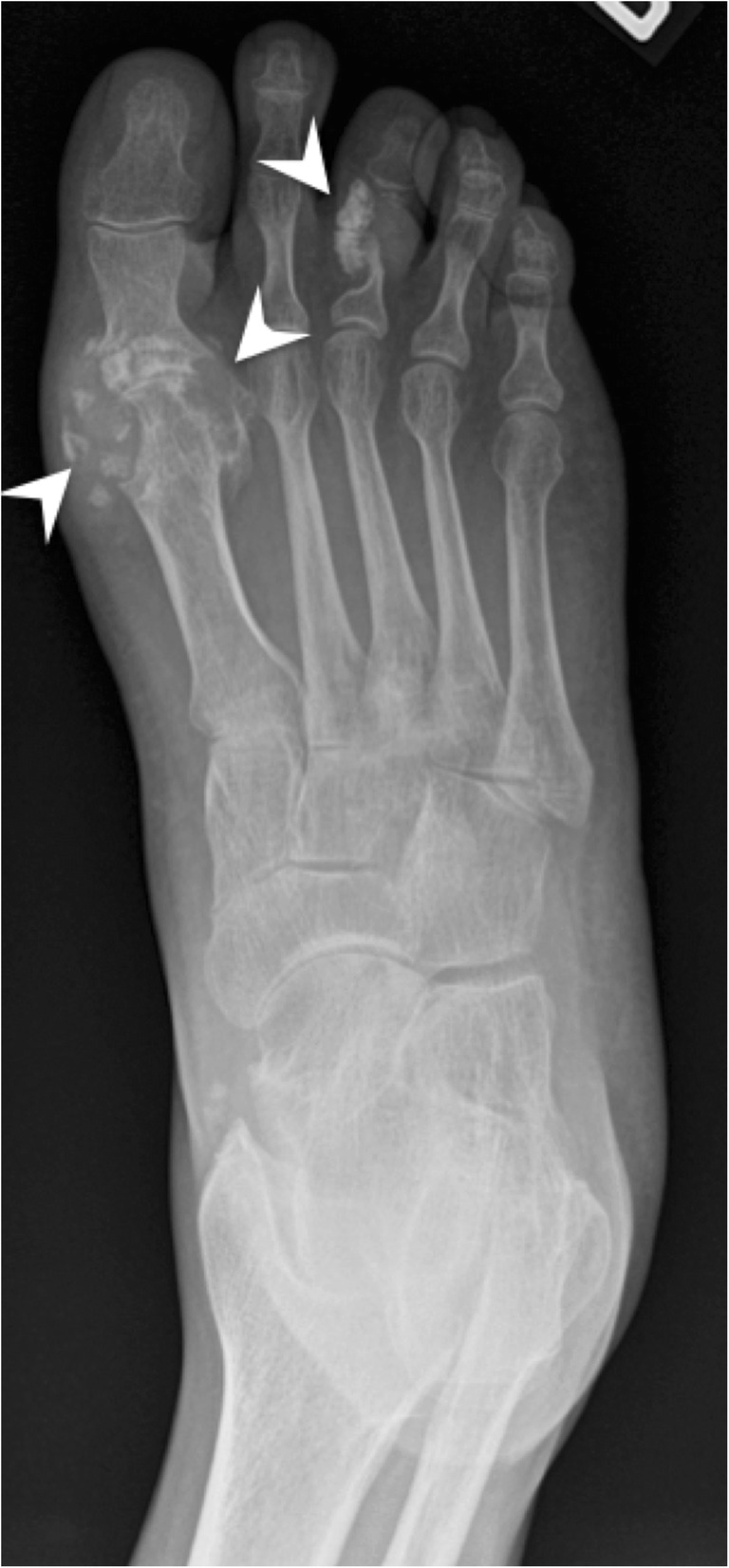
Gout. Frontal radiograph of the right foot shows tophi and erosions. Some tophi may present with mineralisation (arrowheads)

Gout is a frequent crystal-induced arthropathy. However, calcifications are not seen within cartilage on radiographs and it is uncommon to see them in periarticular tophi in the absence of coexisting renal disease [32]. Occasionally, tophi can show some mineralisation on radiographs (Fig. 16). Uric acid crystals can be identified on CT, or even more accurately, on dual-energy CT. One distinctive feature with ultrasound is that crystals can be seen on the surface of the cartilage, rather than within cartilage, as observed with CPPD deposition [33].

#### Synovial osteochondromatosis

**Fig. 17:**
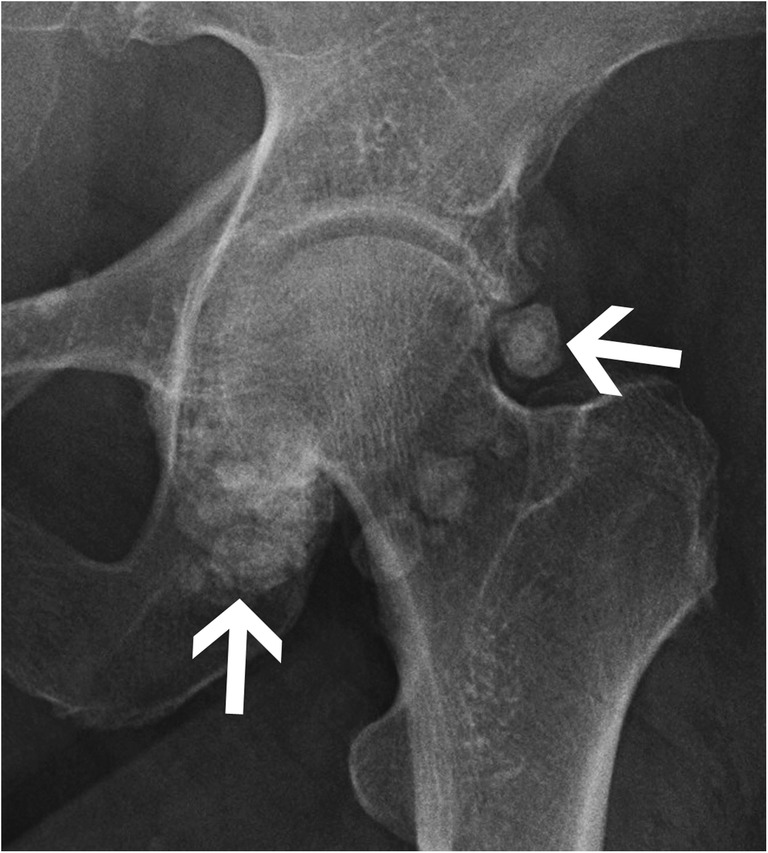
Synovial osteochondromatosis. Anteroposterior radiograph of the left hip shows multiple intra-articular osteocartilaginous bodies (arrows) with ring-like calcifications centred on the coxofemoral joint

Primary synovial osteochondromatosis is a rare metaplastic disorder involving the synovial tissue with proliferation and intra-articular release of osteocartilaginous bodies (Fig. 17). These bodies will usually show typical ring and arc chondroid mineralisation pattern in 70–95% of cases [22, 34]. Marginal pressure erosions and increased joint space are clues to the presence of an intra-articular mass effect and should suggest this diagnosis to the radiologist. More commonly the radiologist will see secondary osteochondromatosis with intra-articular bodies of different sizes in the context of an underlying osteoarthritis. Several rings of calcification may be identified, as opposed to a single ring found in the primary disease. Large bodies usually become ossified and show central adipose tissue on MRI or CT corresponding to the “medullary space” of these osteochondral bodies.

### Intramuscular and subcutaneous calcifications

#### Idiopathic tumoural calcinosis

Idiopathic tumoural calcinosis is a rare inherited disorder also known as Teutschlaender disease [1]. It is more frequent in patients of African descent and manifests in the first and second decades of life. Patients show multiloculated densely calcified periarticular masses caused by abnormal phosphate regulation. Two forms have been described, caused by distinct mutations: one with increased phosphate serum level (generally familial) and one with normal phosphate level (generally sporadic). The massive calcinosis is more often found on the extensor surface of joint in the expected location of bursae [1, 2].

#### Other form of calcinosis (non-idiopathic)

**Fig. 18:**
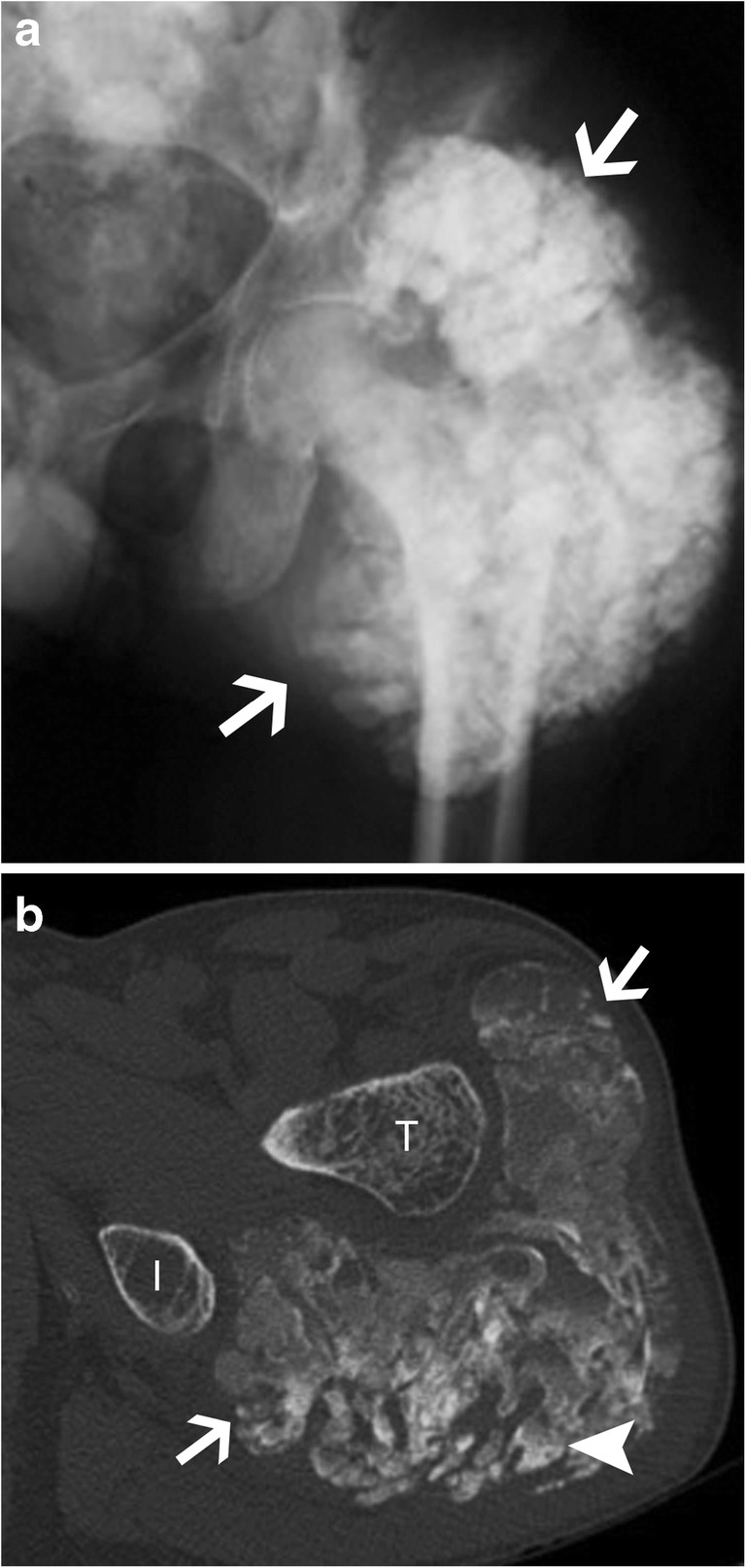
Renal insufficiency with tumoural calcinosis. (**a**) Anteroposterior radiograph of the pelvis and (**b**) axial CT image of the left hip show tumoural calcinosis (arrow) around the left hip with a multiloculated appearance in a clinical context of chronic renal failure. Note fluid-fluid level with denser material layering more posteriorly (arrowhead in b). I: ischium, T: greater trochanter

The most frequent cause of non-idiopathic calcinosis is metabolic (or metastatic) calcifications from chronic renal failure with haemodialysis and renal osteodystrophy (Fig. 18). The periarticular calcified masses are indistinguishable from idiopathic tumoural calcinosis except for bone erosion and destruction that might be seen in this case. It can be associated with vascular calcifications, chondrocalcinosis, bone resorption, osteopenia or osteosclerosis, and tendon pathologies. Other causes of non-idiopathic calcinosis include primary hyperparathyroidism, sarcoidosis, milk-alkali syndrome and hypervitaminosis D. Phosphate serum level will be elevated in any of those settings, with an increase in the phosphocalcic product [22].

**Fig. 19:**
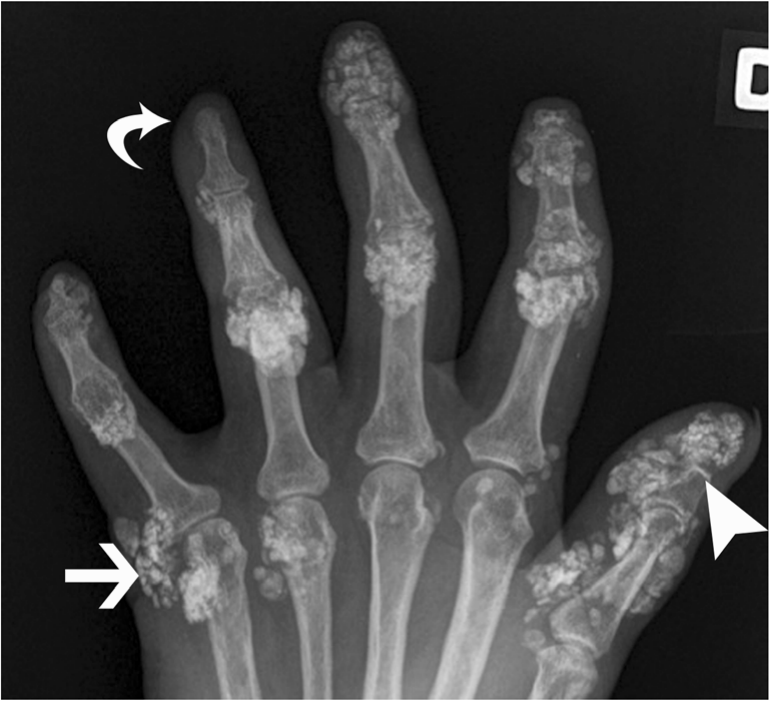
Systemic Sclerosis. Posteroanterior radiograph of the hand shows tumoural calcinosis in the soft tissue (arrow). Note acro-osteolysis (arrowhead) and atrophy of soft tissue (curved arrow), characteristic of systemic sclerosis

**Fig. 20:**
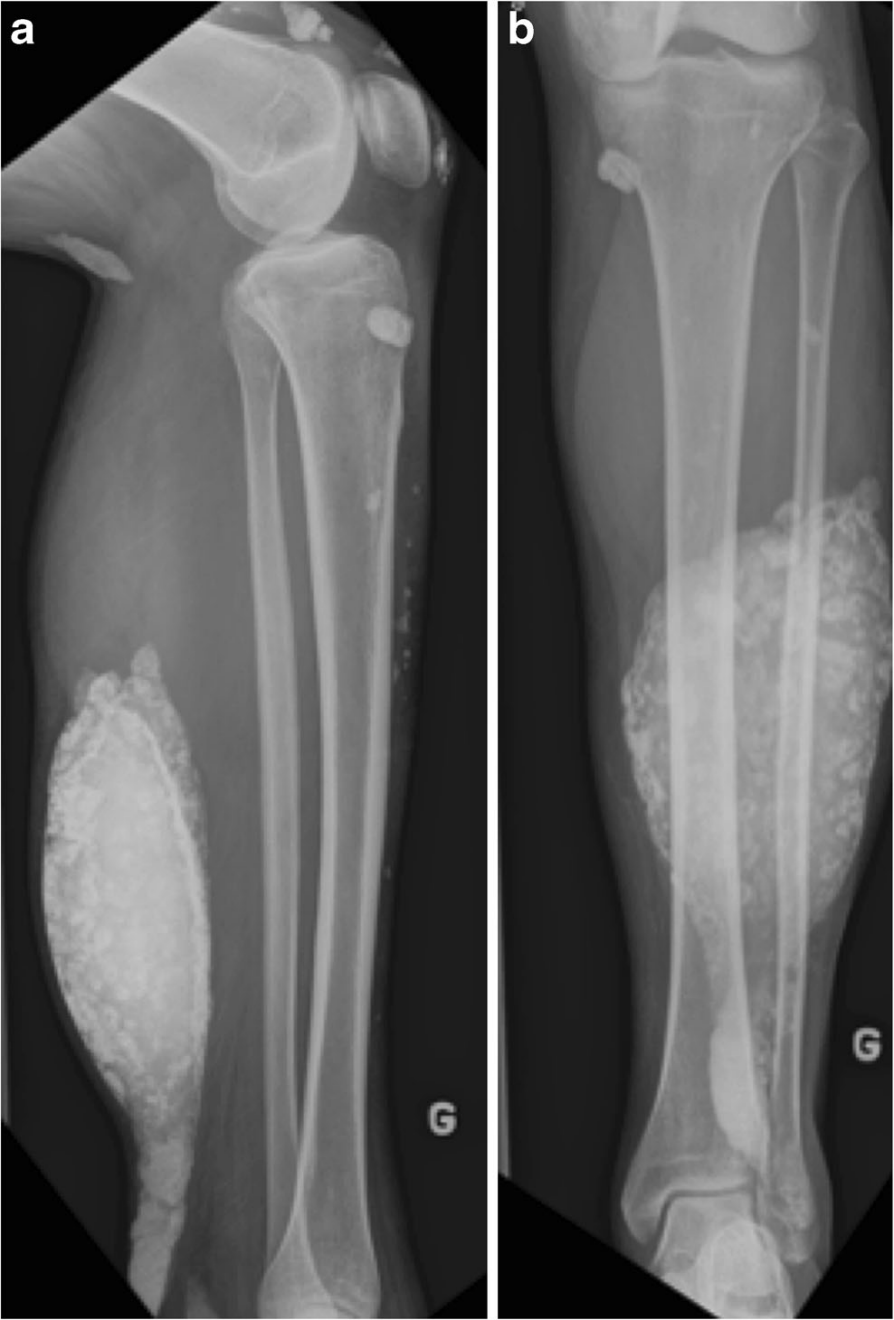
Dermatomyositis. (**a**) Lateral and (**b**) frontal radiographs of the left leg show sheet-like muscular calcifications in a case of dermatomyositis

The second most frequent cause of non-idiopathic calcinosis is collagen-vascular disease. Calcinosis circumscripta is more often seen with progressive systemic sclerosis (scleroderma). It mainly involves the subcutaneous tissues and can cause painful inflammatory dermal papule that can ulcerate and discharge chalky material [35]. The association of acro-osteolysis and skin atrophy with calcinosis is most specific for systemic sclerosis (Fig. 19). Calcinosis universalis is a diffuse calcium deposition in muscles, fascial planes and subcutaneous tissues seen characteristically with dermatomyositis and polymyositis with sheet-like muscle involvement (Fig. 20). Mixed connective tissue disease and, infrequently, lupus erythematosus may show calcinosis as well, although it is less specific [36].

Investigations should include serum calcium and serum phosphate levels and antibody screening for rheumatic disease. Other tests can include other ions, parathormone and vitamin D levels.

#### Vascular calcifications and lesions

**Fig. 21:**
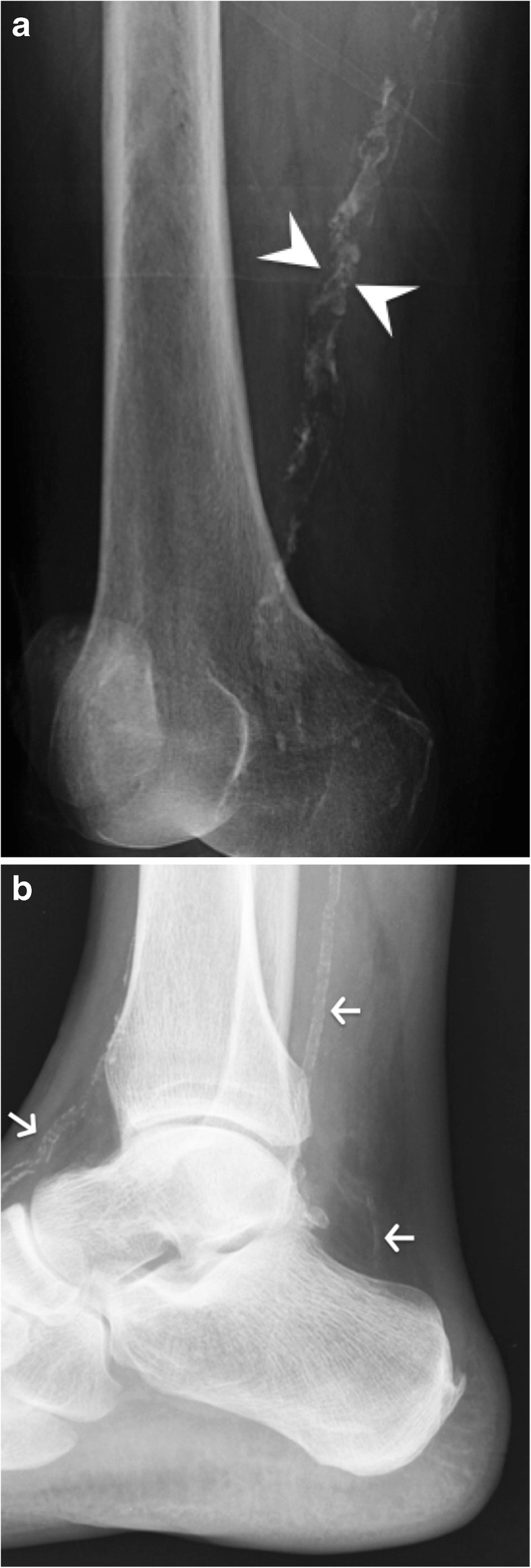
Vascular calcifications. (**a**) Oblique radiograph of the femur shows atherosclerotic vascular calcifications (arrowheads) and (**b**) lateral radiograph of the ankle shows metabolic vascular calcifications (arrows)

Arterial calcification can be either dystrophic or metabolic and will show a “double-tracked” appearance (Fig. 21) [37]. In atherosclerosis, dystrophic calcifications will involve the intima and show a more “chunky” and irregular appearance. On the contrary, metabolic calcifications, such as those seen in chronic renal failure, are more often thin and delicate and found in the media.

**Fig. 22:**
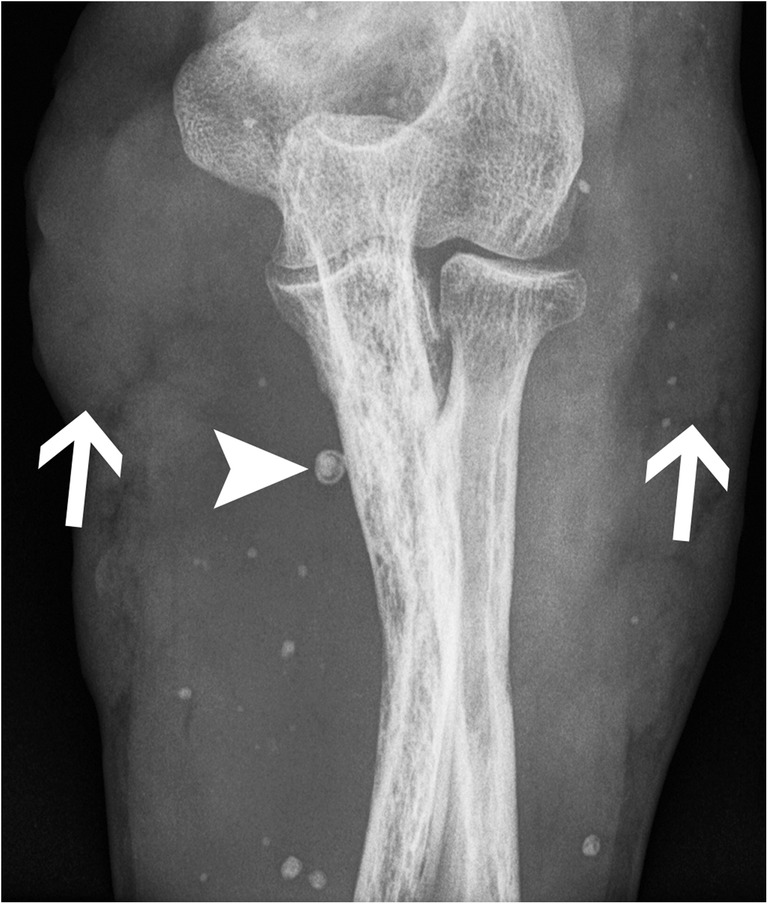
Phleboliths. Anteroposterior radiograph of the elbow shows phleboliths in the soft tissues with the characteristic central lucency (arrowhead) in a patient with a venous malformation of the upper extremity. Note multifocal soft tissue masse effect that corresponds to the diffuse venous malformation (arrows)

Venous calcifications secondary to thrombosis are represented classically by phleboliths, with a focal well-delineated calcification with a denser rim and a central lucency (Fig. 22). It is commonly seen in the pelvis and lower extremities.

Vascular malformations and tumours are now classified following the International Society for the Study of Vascular Anomalies (ISSVA) classification. A venous malformation will demonstrate a soft-tissue mass with occasional phleboliths and, less frequently, with adjacent skeletal anomalies. It can increase in size with Valsalva manoeuvre and be flattened with direct pressure. It usually grows in proportion with the patient or with hormonal stimulation (puberty, pregnancy), but does not regress. Further investigation should include a Doppler US assessment to differentiate from other vascular anomalies [38], showing low venous flow or absence of flow. CT can demonstrate better a phlebolith as well as possible fatty soft tissue components. Additionally, MRI may show fluid-filled cavities and will help determine the extension of the disease in the adjacent tissues.

#### Infections

**Fig. 23:**
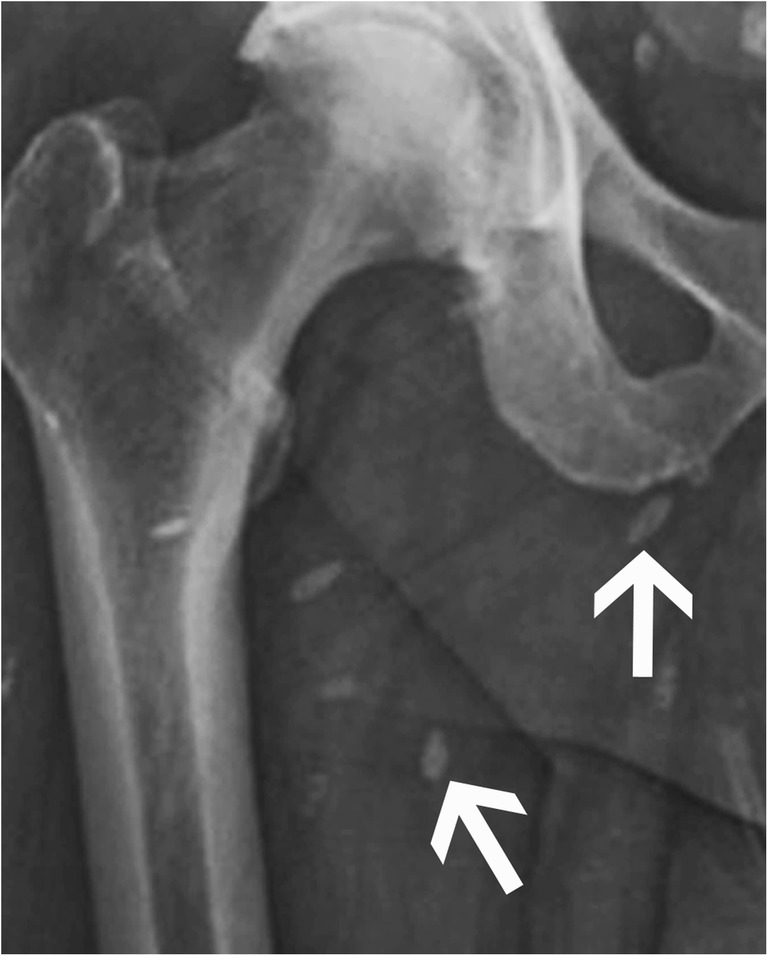
Cysticercosis. Anteroposterior radiograph of the pelvis shows multiple cigar-shaped calcifications characteristic of cysticercosis

Dystrophic calcifications can occur in almost any chronic infectious disease. Previously, psoas calcifications would have strongly suggested presence of spinal tuberculosis with secondary chronic iliopsoas pyomyositis. Nowadays, iliopsoas pyomyositis will be caused mostly by urinary tract or gastrointestinal infections and will not show calcifications [39]. Some typical pattern can suggest a specific diagnosis, such as small “cigar-shaped” intramuscular and subcutaneous calcifications in cysticercosis (Fig. 23), trichinosis, dracunculiasis, and, more rarely, external ear chondral calcifications in syphilis, or nerve calcification in leprosy.

#### Soft tissue tumours

**Fig. 24:**
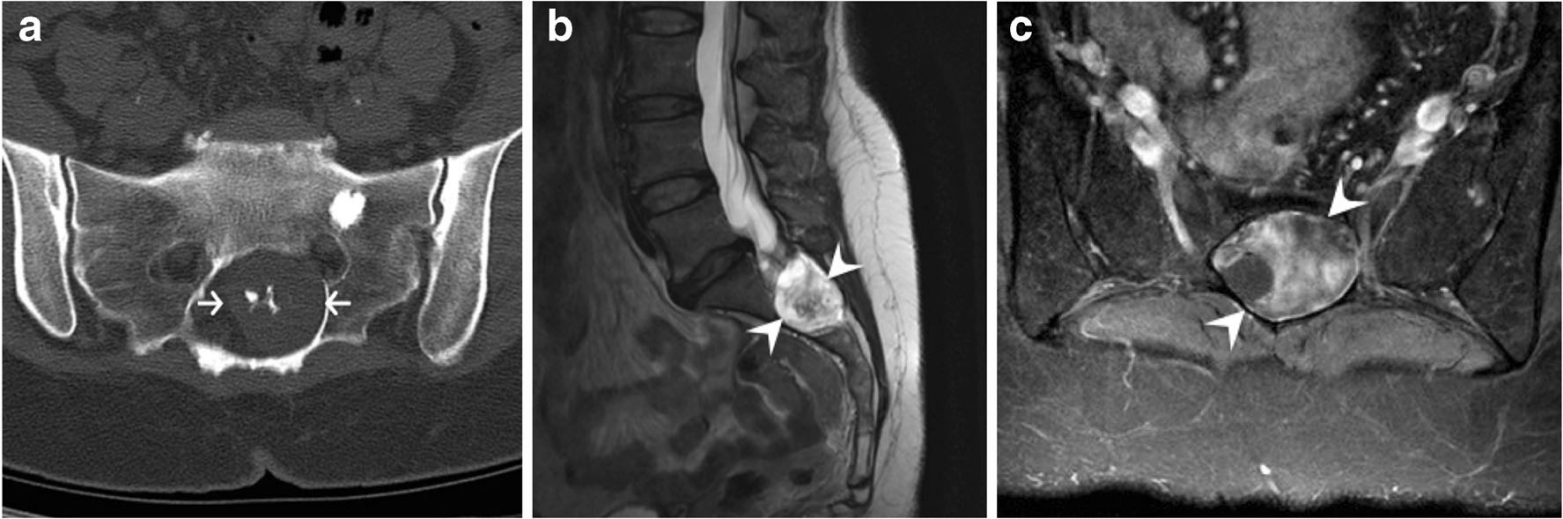
Schwannoma. (**a**) axial CT image of the sacrum shows calcifications in a lesion and (**b**) sagittal T2-weighted MRI image of the sacrum (arrows) and axial fat-saturated T1-weighted MRI image of the sacrum following gadolinium enhancement show the specific signal inside the schwannoma (arrowheads)

Although rare, extraosseous chondromas show a mass effect with typical cartilaginous calcifications in approximately half of cases. Most (82%) involve the hands and feet. Nerve sheath tumours and lipoma are other benign tumours in which calcifications and/or ossifications may rarely occur (Fig. 24) [22].

**Fig. 25:**
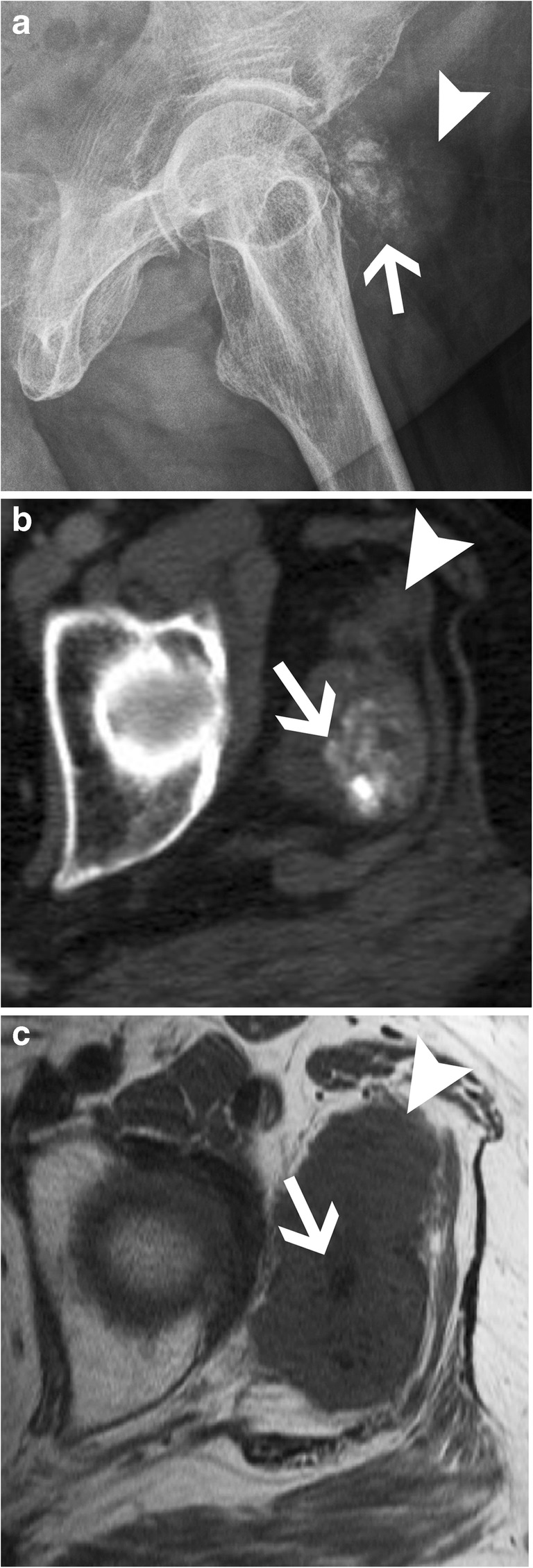
Synovial sarcoma. (**a**) Frog-leg radiograph, (**b**) axial CT image and (**c**) axial T1-weighted image of the left thigh show faint calcifications (arrow in **a**, **b** and **c**) that are located inside a tumour which is better seen on the CT and MRI assessment (arrowhead in **a**, **b** and **c**). Synovial sarcoma was found at pathology

**Fig. 26:**
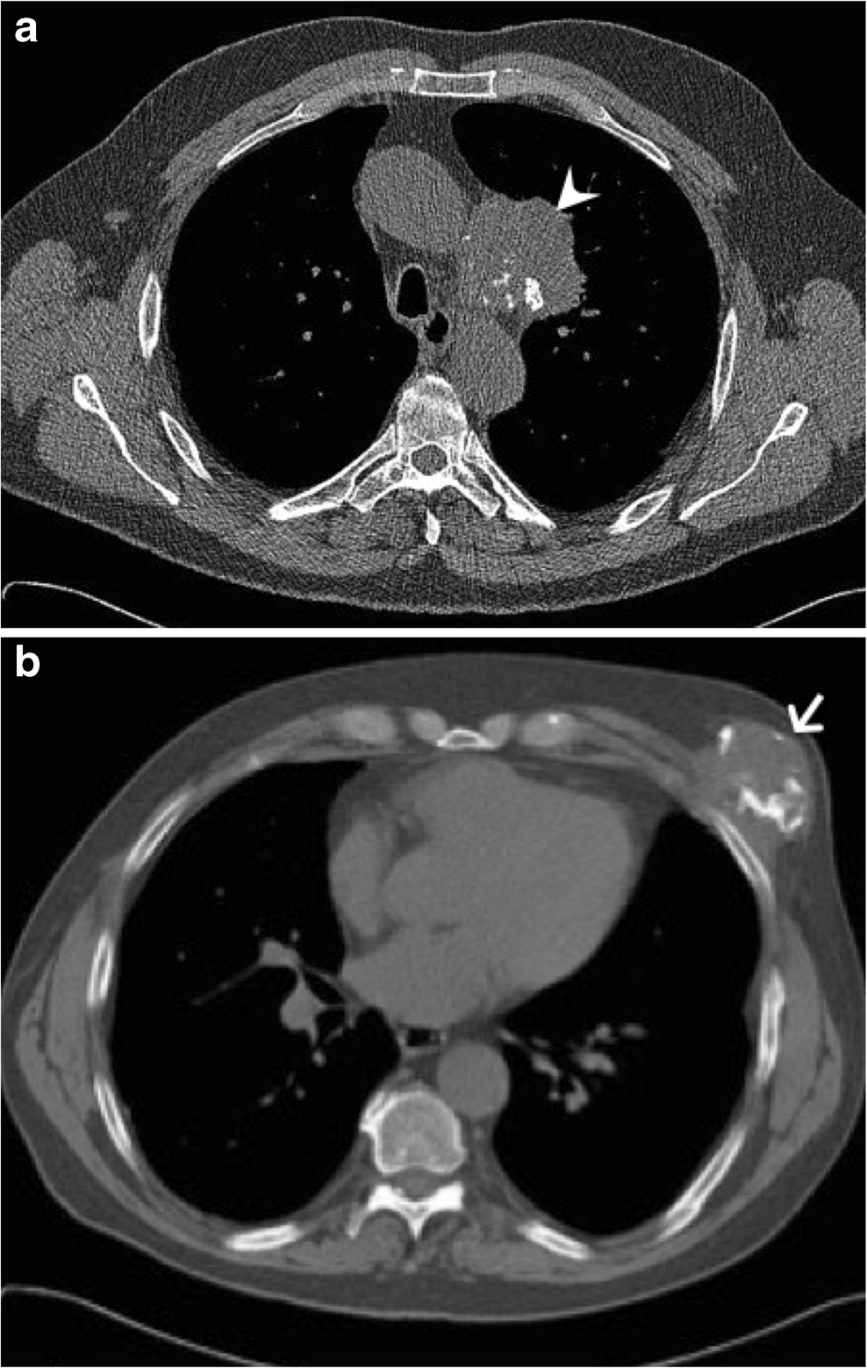
Metastatic calcified lung adenocarcinoma. (**a**, **b**) Two axial CT images of the thorax show calcified lung adenocarcinoma (arrowhead) (**a**) and calcified soft tissue chest wall metastasis (arrow) (**b**)

Synovial sarcoma is the fourth most frequent soft tissue sarcoma, and one-third will exhibit calcifications on radiograph (Fig. 25). In a young adult, the presence of a periarticular mass of the lower extremity showing faint calcifications should raise suspicion for this diagnosis. Extraosseous chondrosarcoma, osteosarcoma, including parosteal osteosarcoma, and metastasis (Fig. 26) can also show calcifications, but are extremely rare [40]. Cross-sectional studies will help in delineating the mass to plan for biopsy and treatment. One helpful clue in differentiating extraosseous osteosarcoma or chondrosarcoma from heterotopic ossification (HO) is that calcification and/or ossification will be more central in sarcoma as opposed to peripheral in HO (Fig. 3).

Remember that tumour necrosis, following either chemotherapy or radiation therapy, may show dystrophic calcifications inside a soft tissue tumour.

Injection site fat necrosis and granulomas are common non-neoplastic tumour-like lesions that tend to calcify and are typically found in expected injection sites, most frequently in the gluteus maximus muscle. Lymph nodes can also show calcifications due to calcifying metastasis (Fig. 26), from adenocarcinoma or medullary carcinoma for example, or granulomatous diseases, caused by sarcoidosis and tuberculosis, for example.

#### Differential diagnosis of intramuscular and subcutaneous calcifications

HO is another non-neoplastic lesion that shows calcifications in the early stages, before the typical bone organisation pattern is recognised (Fig. 3). When occurring in a muscle, it is called myositis ossificans circumscripta, but it can be found in virtually any soft tissue, after trauma. Myositis ossificans can have an aggressive and worrisome clinical presentation and histopathology, suggesting a sarcoma. Follow up study with radiograph or CT is recommended and will show faint calcifications that will eventually evolve towards a focal ossification with a classical zonal distribution (Fig. 3). Burns are a classic cause of heterotopic ossification. Chronic venous insufficiency in the lower extremities will often have heterotopic ossification with a honeycomb appearance that coalesces in the subcutaneous tissues [41]. There are two hereditary forms called progressive osseous heteroplasia and fibrodysplasia ossificans progressive. The latter causes severe ossifications that will eventually prevent adequate motion, causing respiratory failure and death at an early age.

## Conclusion

Calcifications are encountered on imaging by radiologists on a daily basis. An appropriate identification and analysis of those calcifications might be helpful in their practice. In this manuscript, we exposed our two-step method to make a correct diagnosis. First, radiologists have to differentiate between calcification from an ossification or a foreign body. Then, they must correctly identify their location, because it will significantly narrow the differential diagnosis and reduce unnecessary investigations. Hydroxyapatite deposition disease (HADD) is most often responsible for intra-tendinous calcifications and calcium pyrophosphate dihydrate (CPPD) crystal deposition disease, for intra-articular calcifications. A few examples of the most frequent soft tissue calcifications are secondary tumoural calcinosis from renal insufficiency, or collagen vascular diseases and vascular calcifications (arterial or venous).
